# Frequency specific brain networks in Parkinson’s disease and comorbid depression

**DOI:** 10.1007/s11682-016-9514-9

**Published:** 2016-02-05

**Authors:** Long Qian, Yi Zhang, Li Zheng, Xuemei Fu, Weiguo Liu, Yuqing Shang, Yaoyu Zhang, Yuanyuan Xu, Yijun Liu, Huaiqiu Zhu, Jia-Hong Gao

**Affiliations:** 1 Department of Biomedical Engineering, Peking University, Beijing, 100871 China; 2 School of Life Science and Technology, Xidian University, Xi’an, Shaanxi 710071 China; 3 Department of Neurology, Affiliated Brain Hospital of Nanjing Medical University, Nanjing, 210029 China; 4 Department of Biological Sciences, National University of Singapore, Singapore, 119077 Singapore; 5 Center for MRI Research, Academy for Advanced Interdisciplinary Studies, Peking University, Beijing, 100871 China; 6 Beijing City Key Lab for Medical Physics and Engineering, Institute of Heavy Ion Physics, School of Physics, Peking University, Beijing, 100871 China; 7 McGovern Institute for Brain Research, Peking University, Beijing, 100871 China

**Keywords:** Parkinson’s Disease, Depression, Brain Network, Frequency Specificity, Complementary Ensemble Empirical Mode Decomposition

## Abstract

The topological organization underlying the human brain was extensively investigated using resting-state functional magnetic resonance imaging, focusing on a low frequency of signal oscillation from 0.01 to 0.1 Hz. However, the frequency specificities with regard to the topological properties of the brain networks have not been fully revealed. In this study, a novel complementary ensemble empirical mode decomposition (CEEMD) method was used to separate the fMRI time series into five characteristic oscillations with distinct frequencies. Then, the small world properties of brain networks were analyzed for each of these five oscillations in patients (n = 67) with depressed Parkinson’s disease (DPD, n = 20) , non-depressed Parkinson’s disease (NDPD, n = 47) and healthy controls (HC, n = 46). Compared with HC, the results showed decreased network efficiency in characteristic oscillations from 0.05 to 0.12 Hz and from 0.02 to 0.05 Hz for the DPD and NDPD patients, respectively. Furthermore, compared with HC, the most significant inter-group difference across five brain oscillations was found in the basal ganglia (0.01 to 0.05 Hz) and paralimbic-limbic network (0.02 to 0.22 Hz) for the DPD patients, and in the visual cortex (0.02 to 0.05 Hz) for the NDPD patients. Compared with NDPD, the DPD patients showed reduced efficiency of nodes in the basal ganglia network (0.01 to 0.05 Hz). Our results demonstrated that DPD is characterized by a disrupted topological organization in large-scale brain functional networks. Moreover, the CEEMD analysis suggested a prominent dissociation in the topological organization of brain networks between DPD and NDPD in both space and frequency domains. Our findings indicated that these characteristic oscillatory activities in different functional circuits may contribute to distinct motor and non-motor components of clinical impairments in Parkinson’s disease.

**Electronic supplementary material:**

The online version of this article (doi:10.1007/s11682-016-9514-9) contains supplementary material, which is available to authorized users.

## Introduction

Parkinson’s disease is the most common movement disorder, characterized by cardinal motor symptoms, including tremor, rigidity, bradykinesia and postural instability (Aarsland et al. 2012), and is associated with various affective symptoms (Luo et al. 2014; F. M. Skidmore et al. 2013). Depression is considered one of the most commonly observed neuropsychiatric disturbances in Parkinson’s disease, affecting approximately 35% of patients with Parkinson’s disease (Aarsland et al. 2012). However, the pathophysiology of depression underlying Parkinson’s disease remains unclear (McDonald et al. 2003; Postuma et al. 2012; Ravina et al. 2007).

Studies indicate a complex relationship between depression and the motor defect in Parkinson’s disease and that these two aspects may influence separate as well as overlapping neural circuits (McDonald et al. 2003; Postuma et al. 2012; Ravina et al. 2007). Resting state functional connectivity (FC) research further demonstrated the association of convergent and divergent brain connectivity patterns between non-depressed Parkinson's disease (NDPD) and depressed Parkinson's disease (DPD) patients (Luo et al. 2014). Whereas NDPD patients are related with abnormalities in the mesolimbic-putamen circuit, the DPD group is associated with aberrations in the prefrontal-limbic network, temporal-putamen and mesolimbic-putamen circuits. Based on our knowledge, no work has been reported to delineate the topological organizations of whole-brain functional connectivity networks (FCNs) for both patient groups simultaneously.

In the past decades, graph theoretical analysis has been demonstrated to provide a powerful framework for characterizing topological properties of the brain networks (Bullmore and Sporns 2009), which is typically achieved through all major modalities of magnetic resonance imaging (MRI) and neurophysiological data acquisition from both functional and structural perspectives (Bullmore and Bassett 2011). Under this framework, resting state functional MRI is applied to measure the topological organization of brain networks in this current research. Studies using resting state functional MRI (fMRI) have assessed the potential associations between the topological organization of the brain network and cognitive performance, as well as its association with psychiatric brain disorders (Baggio et al. 2014; Itahashi et al. 2014; Yong Liu et al. 2008; Stam et al. 2007; van den Heuvel et al. 2009).

The majority of previous studies concerning the brain network derived from resting state fMRI focused on a low frequency of signal oscillation from 0.01 to 0.1 Hz (Bullmore and Sporns 2009; Van Den Heuvel and Hulshoff Pol 2010). However, the frequency specificities concerning the topological properties of the brain networks have not been fully revealed. In addition, previous studies demonstrated motor and non-motor components of clinical impairment in Parkinson’s disease contributing to different functional circuit oscillatory activities across multiple frequency bands (Brown 2003; Huebl et al. 2011; Li et al. 2012; Neufeld et al. 1994; Oswal et al. 2013; Sinanovic et al. 2005; Soikkeli et al. 1991). Specifically, motor impairments in Parkinson’s disease were associated with a generalized slowing of the electroencephalogram (EEG) and magnetoencephalography (MEG) frequency (Oswal et al. 2013; Soikkeli et al. 1991), an increase of beta frequency waves (Li et al. 2012; Oswal et al. 2013), and abnormal subthalamo-pallidal circuit frequency (<30 Hz and >60 Hz) (Brown 2003; Oswal et al. 2013); non-motor symptoms in Parkinson’s disease such as depression (Huebl et al. 2011) and dementia (Neufeld et al. 1994; Sinanovic et al. 2005) were related to the alterations in alpha and delta rhythms, respectively (Oswal et al. 2013). Nevertheless, the specific BOLD oscillation related to brain networks in NDPD and DPD patients remains to be elucidated.

Derived from the aforementioned literature, it was hypothesized that the functional organizations of brain networks in both patient groups were disrupted. Furthermore, we intended to investigate the association and dissociation in the topological organization of intrinsic brain networks between NDPD and DPD patients from the perspectives of both spatial and spectral spaces. To achieve our goals, a novel data-driven approach named complementary ensemble empirical mode decomposition (CEEMD) was introduced to separate the fMRI time series into several intrinsic mode functions (IMFs) with distinct frequencies. CEEMD can automatically isolate the underlying processes of BOLD activities in a data-driven manner without any assumption of linearity, stationarity, or recourse to any rigid prior chosen band-pass filter, which had been discussed in our previous works (Qian et al. 2015; Song et al. 2014). After applying the CEEMD method, the small world properties of brain networks were analyzed for each of these brain oscillations in both patients and healthy control (HC) groups. Inter-group differences were measured as well.

## Materials and methods

### Participants

The study was approved by Medical Research Ethical Committee of Nanjing Brain Hospital. All patients were recruited from Nanjing Brain Hospital (Nanjing, China) and written informed consents were obtained from all subjects prior to MRI scanning. Seventy patients (37 males) with idiopathic Parkinson’s disease and 50 age- and gender- matched healthy controls (23 males, *P* > 0.05) were recruited. All the subjects were right-handed. The selection of Parkinson’s disease patients fulfilled the criteria of UK Parkinson’s Disease Society Brain Bank for idiopathic Parkinson’s disease (Gibb and Lees 1988). All subjects with Mini-Mental State Examination (MMSE) scores < 24 were also excluded. Patients on dopamine agonists were also excluded, and the dopamine dosing for all patients was stable for at least 4 weeks before and during the study. Laboratory examinations and MRI scans were performed to exclude other diseases. Healthy controls were interviewed to confirm that they had no history of a neurological disorder or psychiatric illness, and no gross abnormality was observed from their brain MRI images (Table 1 and 2).

**Table 1:** Cortical and subcortical regions of interest defined in the study

Index	Region	Abbr.	Index	Region	Abbr.
(1,2)	Precental gyrus	PreCG	(47,48)	Lingual gyrus	LING
(3,4)	Superior frontal gyrus, dorsolateral	SFGdor	(49,50)	Superior occipital gyrus	SOG
(5,6)	Superior frontal gyrus, orbital part	ORBsup	(51,52)	Middle occipital gyrus	MOG
(7,8)	Middle frontal gyrus	MFG	(53,54)	Inferior occipital gyrus	IOG
(9,10)	Middle frontal gyrus, orbital part	ORBmid	(55,56)	Fusiform gyrus	FFG
(11,12)	Inferior frontal gyrus, opercular part	IFGoperc	(57,58)	Postcentral gyrus	PoCG
(13,14)	Inferior frontal gyrus, triangular part	IFGtriang	(59,60)	Superior parietal gyrus	SPG
(15,16)	Inferior frontal gyrus, orbital part	ORBinf	(61,62)	Inferior parietal, but supramarginal and angular gyri	IPL
(17,18)	Rolandic operculum	ROL	(63,64)	Supramarginal gyrus	SMG
(19,20)	Supplementary motor area	SMA	(65,66)	Angular gyrus	ANG
(21,22)	Olfactory cortex	OLF	(67,68)	Precuneus	PCUN
(23,24)	Superior frontal gyrus, medial	SFGmed	(69,70)	Paracentral lobule	PCL
(25,26)	Superior frontal gyrus, medial orbital	ORBsupmed	(71,72)	Caudate nucleus	CAU
(27,28)	Gyrus rectus	REC	(73,74)	Lenticular nucleus, putamen	PUT
(29,30)	Insula	INS	(75,76)	Lenticular nucleus, pallidum	PAL
(31,32)	Anterior cingulate and paracingulate gyri	ACG	(77,78)	Thalamus	THA
(33,34)	Median cingulate and paracingulate gyri	DCG	(79,80)	Heschl gyrus	HES
(35,36)	Posterior cingulate gyrus	PCG	(81,82)	Superior temporal gyrus	STG
(37,38)	Hippocampus	HIP	(83,84)	Temporal pole: superior temporal gyrus	TPOsup
(39,40)	Parahippocampal gyrus	PHG	(85,86)	Middle temporal gyrus	MTG
(41,42)	Amygdala	AMYG	(87,88)	Temporal pole: middle temporal gyrus	TPOmid
(43,44)	Calcarine fissure and surrounding cortex	CAL	(89,90)	Inferior temporal gyrus	ITG
(45,46)	Cuneus	CUN			

The regions are presented according to a prior template obtained from an AAL atlas; odd numbers represent the corresponding brain regions in the left hemisphere, and even numbers denote the specific brain regions in the right hemisphere. AAL: automated anatomical labeling

**Table 2:** Demographic and neuropsychological characteristics of all subjects

	HC (n = 46)	NDPD (n = 47)	DPD (n = 20)	P value
	Mean ± SD	Mean ± SD	Mean ± SD	
Age(years)	57.74 ± 5.56	57.64 ± 7.00	58.05 ± 7.72	0.973*
Education(years)	11.61 ± 4.95	10.83 ± 3.29	11.15 ± 3.12	0.647*
Gender(M/F)	22/24	25/22	9/11	0.791#
HDRS-17	2.17 ± 2.42	6.98 ± 3.29	20.45 ± 4.58	0.0001 ^a, b, c^
UPDRS III	NA	26.21 ± 13.44	27.65 ± 13.17	0.689^@^
H&Y	NA	1.63 ± 0.54	1.43 ± 0.59	0.175^@^
LED(day/mg)	NA	553.69 ± 345.43	500.63 ± 412.41	0.589^@^
Duration time of Parkinson’s disease	NA	6.28 ± 3.35	5.35 ± 2.81	0.282^@^

Values are represented as the mean ± SD. For comparisons of demographics, *P values are obtained using one-way ANOVA tests; #P value for the gender distribution in the three groups was obtained using *χ* ^2^ test. Comparisons of neuropsychological scores among the three groups (HC, NDPD, DPD) were performed using a separate one-way ANOVA. Post hoc pairwise comparisons were performed using *t*-tests. The UPDRS III, H&Y, LED and Duration time of Parkinson's disease were compared utilizing a two sample t-test between NDPD and DPD for ^@^P value. *P* < 0.05 was considered significant

*NA* not applicable, *F* female, *M* male, *HC* healthy control, *NDPD* non-depressed Parkinson's disease, *DPD* depressed Parkinson's disease, *HDRS-17* 17-item Hamilton Depression Rating Scale, *UPDRS III* Unified Parkinson’s Disease Rating Scale motor part III, *MMSE* Mini-Mental State Examination, *LED* levodopa equivalent dose, *SD* standard deviation

^a^ Post hoc paired comparisons showed significant group differences between HC and NDPD

^b^ Post hoc paired comparisons showed significant group differences between HC and DPD

^c^ Post hoc paired comparisons showed significant group differences between NDPD and DPD

For each Parkinson’s disease patient, all psychometric and neurological evaluations were conducted during a practically defined “on” state. The stage of the disease was evaluated by the Hoehn and Yahr (H&Y) staging scale (Hoehn and Yahr 1998); motor disability was evaluated using the Unified Parkinson’s Disease Rating Scale motor part III (UPDRS III) (Vassar et al. 2012); and global cognitive function was evaluated using the Mini-Mental State Examination (MMSE) score (Folstein et al. 1975). The severity of depression in patients was evaluated by the 17-item Hamilton Depression Rating Scale (HDRS-17) for differentiating NDPD from DPD patients (Stebbins and Goetz 1998; Schrag et al. 2007). All patients with no less than 14 points on the HDRS-17 test were considered depressive (Leentjens et al. 2000). Confirmation of the diagnosis of depression was accomplished by an experienced clinical psychiatrist.

### MRI data acquisition

MR images were acquired on a 3T Siemens Verio system (Siemens, Germany). All subjects were instructed to rest with their eyes closed, not to think about anything in particular, or to fall asleep. A gradient-recalled echo-planar imaging (GRE-EPI) pulse sequence was used for acquiring resting state functional images, with the parameters as follows: TR = 2000 ms, TE = 30 ms, flip angle = 90^°^; matrix size = 64 x 64, FOV = 220 x ^220 mm2^, thickness/gap = 3.5 mm/0.6 mm, in-plane resolution of 3.4 mm x 3.4 mm, slices numbers = 31. The scan for resting state fMRI lasted for 280 seconds, containing 140 brain volumes. Thereafter, anatomical images were acquired using a T_1_-FLAIR sequence, with the parameters as follows: TR = 2530 ms, TE = 3.34 ms, flip angle = 7^°^, matrix = 256 × 192, FOV = 256 x ^256 mm2^, thickness/gap = 1.33 mm/0.5 mm, slices numbers = 128.

### Image preprocessing

Images were analyzed using both FMRIB Software Library (FSL: http://www.fmrib.ox.ac.uk/fsl, version 5.0) and Analysis of Functional NeuroImaging (AFNI: http://afni.nimh.nih.gov/afni, version 2011_12_21_1014). Functional images, after excluding the first 5 time points to ensure signal equilibrium, were corrected for temporal differences and head motions. The mean image was acquired by averaging the volumes. Seven subjects with translation or rotation parameters exceeding ± 2 mm or ± 2 degrees were excluded, and no inter-group difference was observed with respect to head translation or rotation (both *P* > 0.05). To enhance the accuracy of normalization, individual structural images were primarily co-registered to the mean functional image, and then we estimated a nonlinear transformation from individual space of the co-registered structural image into MNI152 space. Spatial normalization of the functional image to a standard template (Montreal Neurological Institute) was performed by using the normalization parameters estimated in the last step, resulting in a functional image series of 61 x 73 x 61 voxels (3-mm isotropic voxels). A regression of nuisance variables from the obtained data, including white matter, ventricular signals, global signals and the six motion parameters determined in the realignment procedure, was performed to reduce the influence of motion and unspecific physiological effects. These images were not spatially smoothed as the previous study suggested (Song et al. 2014). A linear trend was regressed out from the time course of each voxel to remove the signal drifts that arose from scanner instability or other causes.

### Empirical mode decomposition

After preprocessing, CEEMD (Yeh et al. 2010) was performed to separate the time series of each voxel into five intrinsic oscillation rhythms with distinct corresponding frequency bands. CEEMD was originated from empirical mode decomposition (EMD) (Huang et al. 1998) and modified from noise-assisted ensemble empirical mode decomposition (EEMD) (Wu and Huang 2009), which resolved the mode-mixing problem and effectively eliminated the residue noise in each IMF (Wu and Huang 2009; Yeh et al. 2010). The detailed algorithm is presented as follows:

The whole procedure of EMD was depicted by Huang (Huang et al. 1998). Apart from most of the data analysis methods, EMD is an adaptive and efficient method to decompose nonlinear and non-stationary biomedical signals by extracting a series of IMFs from the analyzed signal stage by stage (Lin and Zhu 2012). Mathematically, for a real-valued BOLD signal x(t), the standard EMD determines a set of N IMFs(IMF_i_(t)), *i* = 1 to *N*, and a monotonic residue signal r(t), so that

1 $$ \mathrm{x}\left(\mathrm{t}\right)={\sum}_{\mathrm{i}=1}^{\mathrm{N}}{IMF}_{\mathrm{i}}\left(\mathrm{t}\right)+\mathrm{r}\left(\mathrm{t}\right) $$

To ensure that meaningful frequency estimates can be yielded from the time frequency spectra (e.g. no negative frequencies), all the IMFs satisfied the following conditions: the number of zero crossings and the number of extrema equaled or differed at most by one; and the mean value of the upper and lower envelopes defined by the local maxima and local minima was zero at all points. With the above definition of an IMF, all of the signals could be decomposed in the following steps:

1. Identify all of the local extrema;
2. Interpolating all of the minima (resp. maxima) to produce the lower (resp. upper) signal envelope, elow(t) (resp. eup(t));
3. To obtain the local mean time course using the following formula:

2 $$ \mathrm{m}\left(\mathrm{t}\right)=\left[\mathrm{elow}\left(\mathrm{t}\right)+eup\left(\mathrm{t}\right)\right]/2 $$

4) Obtain the “oscillatory mode” from the equation:

3 $$ \mathrm{r}\left(\mathrm{t}\right)=\mathrm{x}\left(\mathrm{t}\right)-\mathrm{m}\left(\mathrm{t}\right) $$

5) If r(t) meets the standard stopping criterion (shifting process only after the IMF condition is achieved for S consecutive times, in the current study S = 3), IMF_i_(t) = r(t) becomes an IMF. Otherwise set x(t) = r(t) and repeat the above steps.
6) To obtain the next IMF, regard the residue r(t) as a new data and repeat the same procedure until r(t) is smaller than a predetermined value, or r(t) becames a monotone function. Repeat step 6 for the former case, and terminate the shifting process for the latter case. Thus, a series of IMFs was obtained.

### Complementary ensemble empirical mode decomposition

The method of complementary ensemble empirical mode decomposition (CEEMD) was originated from EMD invented by Huang (Huang et al. 1998) and extended from ensemble empirical mode decomposition (EEMD) by Wu (Wu and Huang 2009). The EEMD generates an ensemble of data sets by adding different realizations of white noise with finite amplitude *ε*_0_ to the original data. EMD analysis is then applied to each data series of the ensemble; ultimately, the IMFs are achieved by averaging the respective components in each realization over the ensemble (Wu and Huang 2009). The averaging effect of the assisted white noise ε_f_ will decrease as:

4 $$ {\varepsilon}_f=\varepsilon /\sqrt{NE} $$

In Eq. (4), ε = ε_0_std(y_0_), ε_0_ is the input noise level, y_0_ represents the input signal, and NE is the ensemble number. Theoretically, NE will approach infinity in order to smooth out the assisted white noise. In practice, ε_0_ is chosen in the interval of 0.1-0.4; NE of the order of 100 will generally produce satisfactory results and render the residual noise less than a fraction of 1% of the error (Wu and Huang 2009). In our previous study (Qian et al. 2015), we discussed the selection of parameter *ε*_0_ . Here, we followed it (*ε*_0_ = 0.4; NE = 100). To further reduce the final white noise residue in each IMF component and time consumption, a new method, CEEMD, is applied here, where white noise is particularly included in pairs to the original data (i.e. one positive and one negative) to generate two sets of ensemble IMFs (Yeh et al. 2010).

### Hilbert weighted frequency

After the decomposition step, the Hilbert weighted frequency (HWF) of each IMF (Xie and Wang 2006) was applied to reflect the mean oscillation frequency of the IMF in order to visualize the frequency distribution of each IMF in each group (Song et al. 2014). The detailed algorithms were calculated as follows:

Identify all local extrema;

1. For each IMF in Eq. (1), Hilbert transform was applied using the following formula:

5 $$ {\mathrm{y}}_{\mathrm{i}}\left(\mathrm{t}\right)=\frac{1}{\uppi}\mathrm{P}\int \frac{IMF_{\mathrm{i}}\left(\mathrm{t}^{\prime}\right)}{\mathrm{t}-\mathrm{t}^{\prime }}dt $$

where P indicates the Caushy principle value.

2) Calculate the corresponding analytical signal*z*_*i*_(*t*):

6 $$ {\mathrm{z}}_{\mathrm{i}}\left(\mathrm{t}\right)={IMF}_{\mathrm{i}}\left(\mathrm{t}\right){+iy}_{\mathrm{i}}\left(\mathrm{t}\right)={\mathrm{a}}_{\mathrm{i}}\left(\mathrm{t}\right){\mathrm{e}}^{{\hbox{-} i\theta}_{\mathrm{i}}\left(\mathrm{t}\right)} $$

Where $$ {\mathrm{a}}_{\mathrm{i}}\left(\mathrm{t}\right)=\sqrt{IMF_{\mathrm{i}}^2\left(\mathrm{t}\right)+{\mathrm{y}}_{\mathrm{i}}^2\left(\mathrm{t}\right)} $$ and $$ {\uptheta}_{\mathrm{i}}\left(\mathrm{t}\right)= \arctan \left(\frac{{\mathrm{y}}_{\mathrm{i}}\left(\mathrm{t}\right)}{IMF_{\mathrm{i}}\left(\mathrm{t}\right)}\right) $$

3) The instantaneous frequency of each IMF is defined as:

7 $$ {\mathrm{w}}_{\mathrm{i}}\left(\mathrm{t}\right)=\frac{{d\theta}_{\mathrm{i}}\left(\mathrm{t}\right)}{dt} $$

4) The Hilbert weighted frequency (HWF) of each IMF with m data points is defined as:

8 $$ {HWF}_{\mathrm{i}}=\frac{\sum_{\mathrm{i}=1}^{\mathrm{m}}{\mathrm{w}}_{\mathrm{i}}\left(\mathrm{t}\right){\mathrm{a}}_{\mathrm{i}}^2\left(\mathrm{t}\right)}{\sum_{\mathrm{i}=1}^{\mathrm{m}}{\mathrm{a}}_{\mathrm{i}}^2\left(\mathrm{t}\right)} $$

**Fig. 1:**
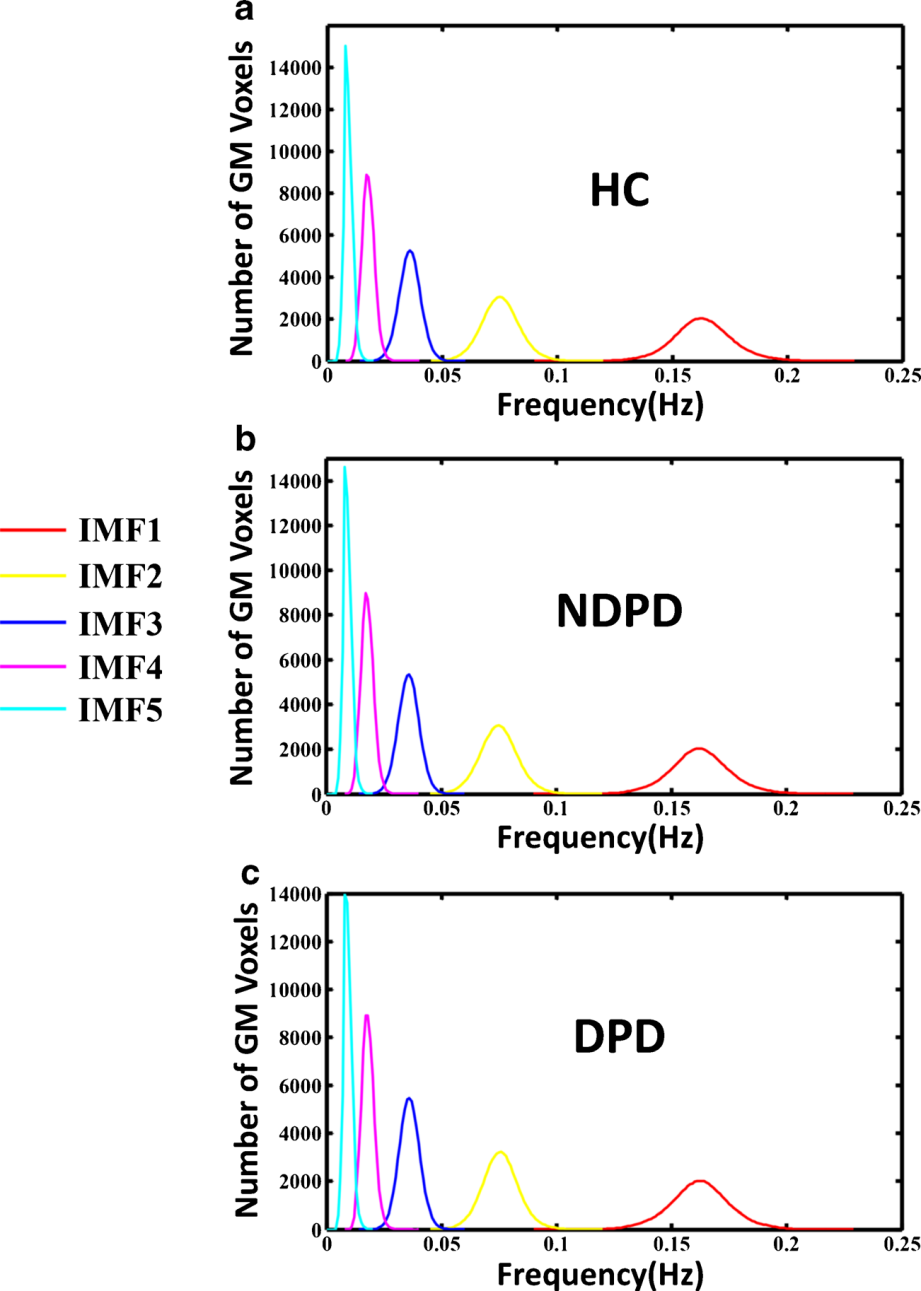
Histogram of frequency distribution among three groups. The histograms of the HWF distributions display the first five IMFs of the voxels in the whole brain gray matter using the CEEMD approach across all subjects within each group. Colors were assigned in the sequence of red, yellow, blue, magenta and cyan from IMF1 to IMF5. Heights of the histograms represent the amount of voxels with HWF equals the frequency on the horizontal axis. Each of the five histograms from Fig. 1a to Fig. 1c represents statistics of the whole-brain gray matter voxels within the HC, NDPD and DPD group respectively. The frequency bands denoted by IMFs were similar within each of the three groups. From Fig. 1a to Fig. 1c, the frequency of each IMF fell into a unique frequency band, with the first IMF (IMF1) indicating the highest frequencies (0.12-0.22 Hz), IMF2 from 0.05 to 0.12 Hz, IMF3 from 0.02 to 0.05 Hz, IMF4 from 0.01 to 0.03 Hz, and IMF5 being the lowest frequency band from 0 to 0.02 Hz. *HWF* Hilbert weighted frequency; *CEEMD* Complementary Ensemble Empirical Mode Decomposition; *IMF* Intrinsic mode decomposition; *HC* healthy control; *NDPD* non-depressed Parkinson's disease; *DPD* depressed Parkinson's disease

For most voxels, the decomposition of the time course yielded only four to five IMFs and the frequency range of the first five IMFs covered 0-0.25 Hz (TR = 2 s). Consequently, only the first five IMFs of each voxel were considered in the current study, denoted as IMF1 to IMF5, and we calculated the HWFs of IMF1 to IMF5 for each voxel to get the histograms of the HWF distribution of IMF1 to IMF5 for the voxels in the whole brain (Fig. 1)

### Graph analysis

#### Network construction

In the current study, Pearson’s correlation was performed to estimate the IMF dependent correlations between each pair of the 90 cortical and subcortical (90 ROI from the common AAL atlas) BOLD signals derived from each individual set. A set of five (90 x 90) inter-regional Pearson’s correlation matrices was then obtained for each subject. Typical graph analyses of the weighted networks ignored negative ties while a recent study proposed to incorporate negative weights into analyses of subgraph detection (Power et al. 2011). Here, we followed the traditional approach. False discovery rate (FDR) correction was applied to regulate the expected FDR at a statistical significance threshold of *P* < 0.05 across ROI pairs within each subject. Thus, five frequency dependent population-based functional connectivity networks were constructed by capturing the underlying common connectivity pattern of the brain at each IMF for each subject in all groups.

#### Network analysis

##### Small-world analysis

Previous studies (He et al. 2008; Liu et al. 2012) demonstrated the two key metrics applied to describe the complex networks in the human brain: clustering coefficient (CP) and characteristic path length (Lp). In order to investigate the small-world properties, Cp and Lp were compared with the corresponding random networks (Maslov and Sneppen 2002). A small-world network possessed a significantly higher clustering coefficient value and similar path length than a random network, that is *γ* = *Cp*(*real*)/*Cp*(*rand*) > 1, *λ* = *Lp*(*real*)/*Lp*(*rand*) ≈ 1 (Watts and Strogatz 1998). In order to observe these frequency specificities of brain networks, graph characteristics were calculated at multi-sparsity (or density), which represented the fraction of the present connections to all possible connections (Watts and Strogatz 1998). Notably, in the current study, the estimation of all parameters was under the consideration of a weighted coefficient, which was consistent with a previous study (Zhang et al. 2011). The estimation of all parameters was calculated using the code provided in the Brain Connectivity Toolbox (BCT) (Rubinov and Sporns 2010).

##### Regional nodal characteristics

To determine the nodal (regional) characteristics of frequency specific brain networks, the regional efficiency (${E}_i^w$) was computed in the present study, which was defined as the inverse of the mean harmonic shortest path length between the target node and all other nodes in the network (Bai et al. 2012; Liu et al. 2012). Considering the sparsity-dependent regional nodal characteristics, the area under the curve of ${E}_i^w$ across a range of the interested sparsity thresholds (S_min_(0.16):0.01:S_max_(0.35)) was regarded as the estimation for each node, denoted as ${E}_i^{\left(w,auc\right)}$, where S_min_ represented a minimum network sparsity in which all nodes would become fully connected in the five IMF-dependent brain networks (Qian et al. 2015)General speaking, the nodes demonstrating high ${E}_i^{\left(w,auc\right)}$ were considered as the hubs (Hosseini et al. 2012). However, in the current study, to integrate the ${E}_i^{\left(w,auc\right)}$ in each frequency band, nodes were first ranked by their efficiencies in descending order in each frequency band, which resulted in five rank orders for each node. Then, the overall rank order (ORO) for each node was obtained by averaging these five rank orders for the specific node, thereafter, the ORO was normalized by dividing the maximal ORO of the network. Specifically, nodes were considered as hubs if their nodal normalized ORO (NORO) was at least one standard deviation (SD) smaller than the average nodal NORO of the network. Additionally, for reference, hubs defined by the conventional method (Zhang et al. 2011) were also presented in the current study.

### Statistical analysis and correlation with clinical variables

To test the differences in age, education level and neuropsychological scores across the three groups, the data was analyzed using separate one-way ANOVAs. Post-hoc pair wise comparisons were then performed using a *t*-test. The gender difference were also analyzed using a χ^2^ test. Pair-wise comparisons were performed using a general linear model to determine the inter-group differences in global network measurements and regional efficiency. The effects of age, gender and years of education were adjusted for all of these analyses. A value of *P* < 0.05 was considered statistically significant except for when comparing the group effects of regional topological characteristics (statistical significance level at *P* < 0.01). Thus, some nodes may show significant differences in several brain oscillation rhythms between each paired comparisons across the three groups. For simplicity, the maximum inter-group significant difference of one node across five IMFs was reported specifically.

To investigate the clinical relevance of altered brain network regional topology in DPD, the correlations of scores of the 17-item Hamilton Depression Rating Scale (HDRS-17) and nodal efficiency (${E}_i^{\left(w,auc\right)}$) were calculated. Pearson’s correlation analysis was applied to control underlying cofounders such as gender, age, and education level (*P* < 0.05).

## Results

### Neuropsychological test results

Twenty patients were diagnosed with DPD according to their HDRS-17 scores. There was no observed inter-group significant difference in age (F = 0.0275, *P* = 0.973), education level (F = 0.4369, *P* = 0.647) or gender (χ^2^ = 0.470, *P* = 0.791). The HDRS-17 scores varied significantly across the three groups (F = 248.16, *P* < 0.0001). No significant difference existed in UPDRS III (t = -0.4029, *P* = 0.6884), H&Y (t = 1.3729, *P* = 0.1745), LED (t = 0.5429, *P* = 0.5890) or Parkinson’s disease duration (t = 1.0843, *P* = 0.2822) between NDPD and DPD groups, as presented in Table 2. The inter-gender difference for HDRS-17 scores was not statistically significant in either the NDPD group (male: 6.92 ± 3.417; female: 6.62 ± 2.599; *P* = 0.757) or the DPD group (male: 20.56 ± 6.044; female: 20.36 ± 3.264; *P* = 0.933), and depression severity was not correlated with age in the NDPD group (R = 0.085, *P* = 0.051).

### Frequency properties of IMF in NDPD, DPD and HC

The histogram of HWF distribution presented in Fig. 1 demonstrated the first five IMFs (IMFs, s = 1, 2, 3, 4 or 5) of voxels determined using the CEEMD method in the whole brain grey matter across all subjects within each groups. The result showed that the same IMF derived from all voxels fell approximately into the same frequency band, where the five IMF components covered a frequency band ranging from 0 to 0.22 Hz [27]. Moreover, the frequency bands denoted by IMFs were similar across the three groups. As demonstrated from Fig. 1a to Fig. 1c, the first IMF (IMF1) indicated the highest frequencies of 0.12-0.22 Hz, IMF2 of 0.05 to 0.12 Hz, IMF3 of 0.02 to 0.05Hz, IMF4 of 0.01 to 0.03 Hz, and IMF5 of 0 to 0.02 Hz as the lowest frequency band. The results suggested that CEEMD could adaptively decompose the original time series into several similar intrinsic oscillatory modes within distinct frequency bands across the three groups.

### Frequency specific global topology alterations in NDPD and DPD

**Fig. 2:**
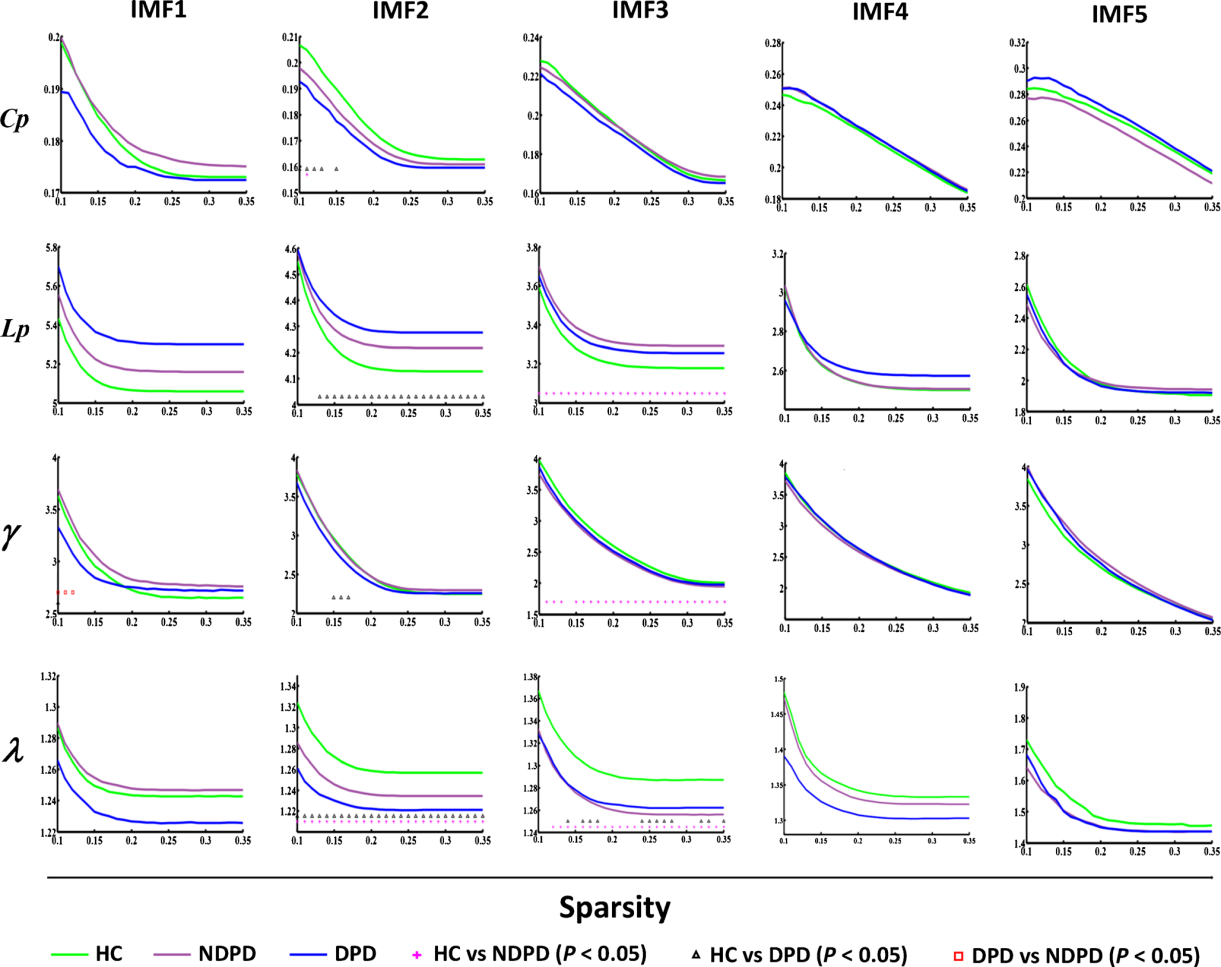
Global measures of frequency specific brain networks were quantified in the HC, NDPD and DPD patients. All groups showed a small-world organization of these five frequency specific FNCs. The significant inter-group overall global topological differences are presented in IMF2 and IMF3. The data points are marked with a red plus sign to indicate a significant difference (HC vs. NDPD; *P* < 0.05), and with a black asterisk to indicate a significant difference (HC vs. DPD; *P* < 0.05). *IMF* Intrinsic mode decomposition; *HC* healthy control; *NDPD* non-depressed Parkinson's disease; *DPD* depressed Parkinson's disease

The significant inter-group global topological difference was observed in IMF1, IMF2 and IMF3 components (Fig. 2), but not in other frequency bands. In addition, all groups showed a small-world organization of these five frequency specific FNCs (Fig. 2).

Fig. 2 shows the mean values of Cp, Lp, γ and λ as a function of sparsity for all groups, respectively. The network metrics were calculated for each individual frequency specific FC graph, while the group mean values and significantly different levels were displayed.

Within IMF1, a significant inter-group difference of γ was observed at a narrow range of sparsity (from 0.1 to 0.12). Within the IMF2, a significantly higher Lp was observed in the DPD group over a wide range of densities as compared to HC (Fig. 2). Both the NDPD and DPD groups showed a significantly lower λ at a range of the density threshold from 0.1 to 0.35. The same network parameters were compared and presented within IMF3 in Fig. 2. Among which, a significantly lower γ and higher Lp were observed in the NDPD group over a wide range of densities relative to HC. Both the NDPD and DPD groups further showed a significantly lower λ over a wide range of densities compared with HC.

### Nodal characteristics

#### Identification of network hubs

**Fig. 3:**
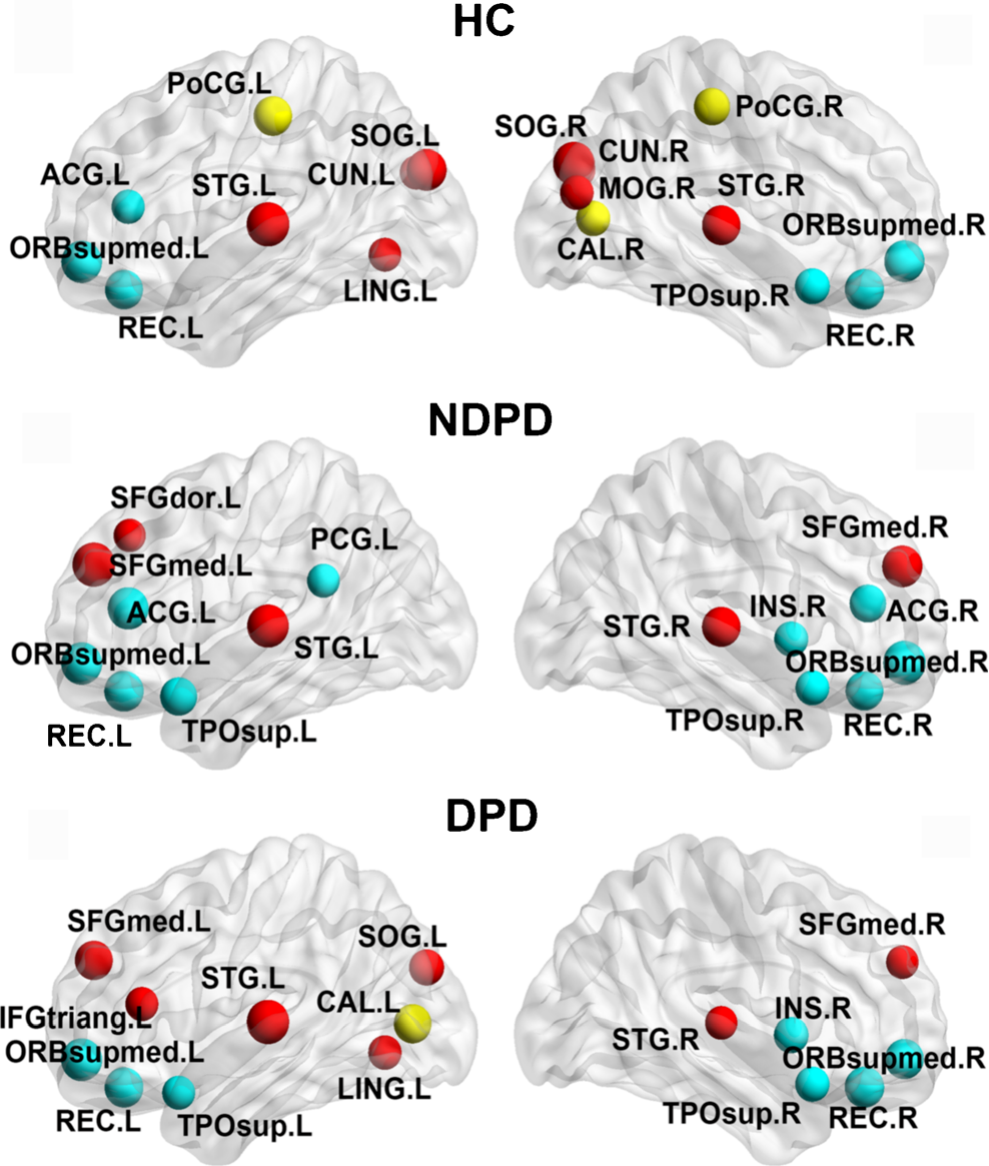
The distribution of hub regions in the HC, NDPD and DPD groups. Hub regions were visualized using BrainNet view (NKLCNL, Beijing Normal University). Three-dimensional rendering maps showed the hub regions defined by normalized overall rank order for each node (Table 3). The hub nodes are colored in red, yellow, and cyan indicating Associations, Primary, Paralimbic regions, respectively. The abbreviations of the regions are shown in Table 1. *HC* healthy control; *NDPD* non-depressed Parkinson's disease; *DPD* depressed Parkinson's disease

Fig. 3 and Table 3 show the nodes identified as hubs. In line with previous reports (Achard et al. 2006; Zhang et al. 2011; Nijhuis et al. 2013; Tomasi and Volkow 2011), several common nodes were observed to present the network hub property across the three groups. In particular, seven of the hub regions were identified for all groups including bilateral medial orbital parts of superior frontal gyrus (ORBsupmed), rectus gyrus (REC), superior temporal gyrus (STG) and right temporal pole part of superior temporal gyrus (TPOsup). Four hub regions: bilateral medial parts of superior frontal gyrus (SFGmed), left TPOsup and right insula (INS), were identified as hub regions in both patient groups, but not in the HC group. Instead of the NDPD group, hub regions in the occipital lobe were only observed in HC and DPD groups—those were the left superior occipital gyrus (SOG) and lingual gyrus (LING) in both the HC and DPD groups, and the bilateral cuneus (CUN), right SOG, calcarine fissure (CAL) and middle occipital gyrus (MOG) in the HC group, and left CAL in the DPD group. Bilateral postcentral gyri (PoCG) were identified as hub regions in the HC group, but not in the other two groups. Hubs in the cingulate gyrus were demonstrated in the HC and NDPD groups—left anterior cingulate gyrus (ACG) in both HC and NDPD groups, left posterior cingulate gyrus (PCG) and right ACG in the NDPD group—but no hub in the cingulate gyrus for the DPD group. In addition, the left dorsal superior frontal gyrus (SFGdor) and triangular part of inferior frontal gyrus (IFGtriang) were identified as a hub in the NDPD and DPD group, respectively. Moreover, convergent results between the hubs were defined by both NORO (Fig. 3 and Table 3) and the conventional method (Supplementary Fig. 1, Supplementary Tables 1 to 5) (Zhang et al. 2011). The hubs identified in the NDPD group were predominantly detected in regions of paralimbic cortices. Similar findings have been reported in a previous study of temporal lobe epilepsy, where the authors also observed a higher proportion of paralimbic hubs in patients than in controls (Zhang et al. 2011).

**Table 3:** Hub regions in HC, NDPD and DPD groups

	Hub regions	Functional classification	Normalized ORO
HC	STG.L	Association	0.0473
	SOG.R	Association	0.0709
	ORBsupmed.L	Paralimbic	0.0898
	ORBsupmed.R	Paralimbic	0.0993
	SOG.L	Association	0.1064
	REC.R	Paralimbic	0.1229
	PoCG.L	Primary	0.1418
	STG.R	Association	0.1418
	REC.L	Paralimbic	0.1489
	PoCG.R	Primary	0.1915
	CUN.R	Association	0.2080
	TPOsup.R	Paralimbic	0.2222
	CAL.R	Primary	0.2364
	MOG.R	Association	0.2506
	ACG.L	Paralimbic	0.2577
	CUN.L	Association	0.2600
	LING.L	Association	0.2695
NDPD	SFGmed.L	Association	0.0437
	ORBsupmed.R	Paralimbic	0.0506
	STG.L	Association	0.0713
	ORBsupmed.L	Paralimbic	0.0851
	ACG.L	Paralimbic	0.0874
	SFGmed.R	Association	0.0989
	REC.L	Paralimbic	0.1195
	STG.R	Association	0.1379
	REC.R	Paralimbic	0.1540
	TPOsup.L	Paralimbic	0.1747
	ACG.R	Paralimbic	0.1816
	INS.R	Paralimbic	0.2391
	TPOsup.R	Paralimbic	0.2460
	PCG.L	Paralimbic	0.2667
	SFGdor.L	Association	0.2782
DPD	STG.L	Association	0.0566
	ORBsupmed.L	Paralimbic	0.0792
	REC.R	Paralimbic	0.1154
	REC.L	Paralimbic	0.1312
	ORBsupmed.R	Paralimbic	0.1335
	SFGmed.L	Association	0.1493
	CAL.L	Primary	0.1629
	SOG.L	Association	0.2172
	INS.R	Paralimbic	0.2240
	TPOsup.R	Paralimbic	0.2376
	LING.L	Association	0.2557
	IFGtriang.L	Association	0.2624
	TPOsup.L	Paralimbic	0.2647
	SFGmed.R	Association	0.2738
	STG.R	Association	0.2828

Major “hubs” of the brain networks in each group defined by normalized overall rank order. The cortical regions were classified as primary, association, and paralimbic. *ORO* overall rank order. For the abbreviations of the regions, refer to Table 1

#### Between-group difference in regional efficiency

**Fig. 4:**
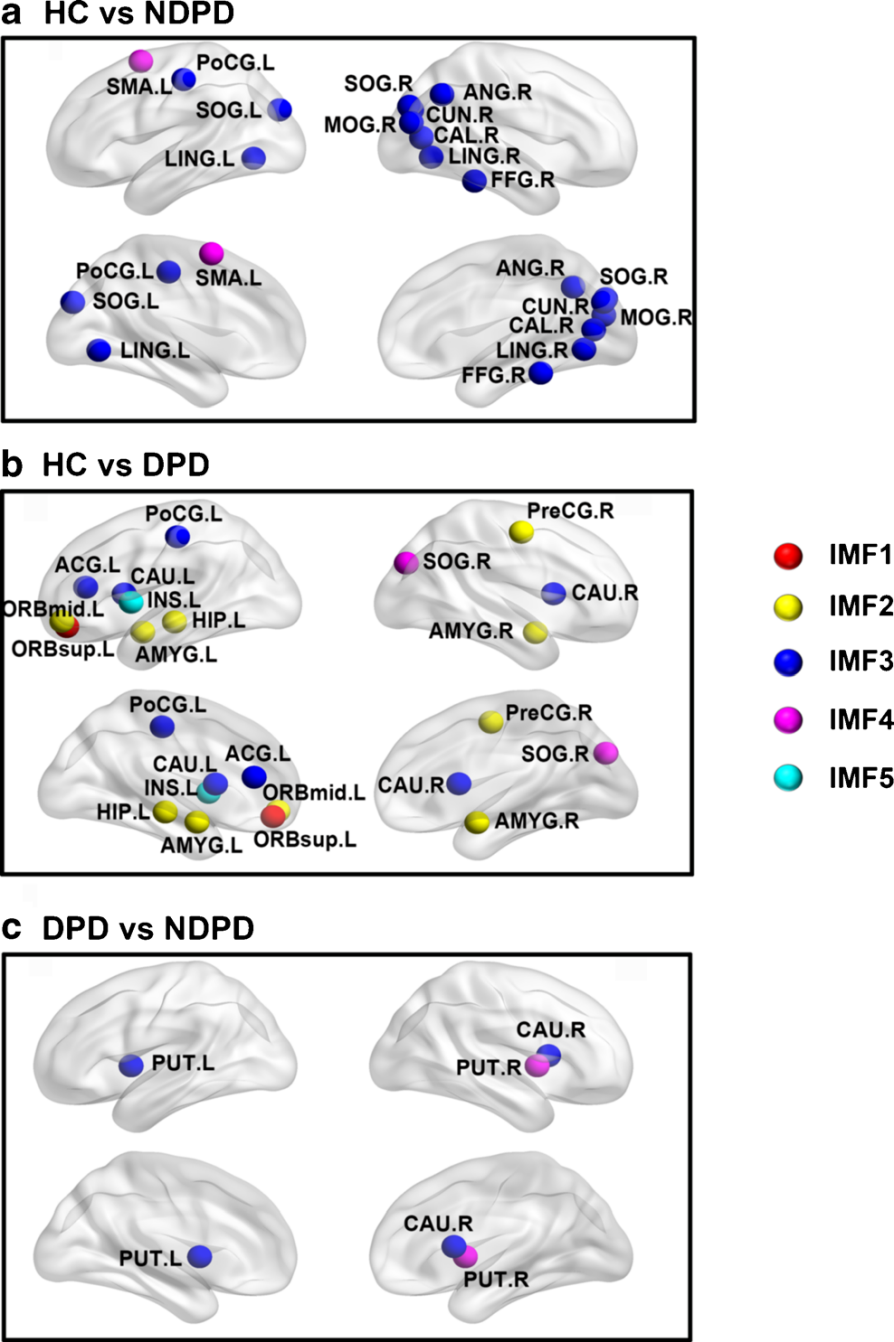
The distribution of brain regions with most significant differences in nodal efficiency among the HC, NDPD and DPD groups across five frequency bands. Nodes from Fig. 4a to Fig. 4c represent the brain regions with the most significant differences across the three groups in the regional efficiencies across five identical frequency bands (*P* < 0.01, uncorrected). Colors were assigned in the sequence of red, yellow, blue, magenta and cyan from IMF1 to IMF5. The nodes with significant topological alterations were mainly distributed in the visual cortex in NDPD patients, and in the paralimbic-limbic and basal ganglia networks in DPD patients. The significant disrupted nodal topological characteristic was dominated in the frequency bands from 0.02 to 0.05 Hz in the visual cortex in the NDPD group, as well as from 0.01 to 0.05 Hz in basal ganglia and from 0.02 to 0.22 Hz in the paralimbic-limbic network in the DPD group. Brain regions were visualized using the BrainNet viewer (NKLCNL, Beijing Normal University). For the abbreviations of the regions, refer to Table 1. *IMF* Intrinsic mode decomposition; *HC* healthy control; *NDPD* non-depressed Parkinson's disease; *DPD* depressed Parkinson's disease

Along with the discovery of a disrupted frequency-specific global network organization, pair-wise group comparisons on regional efficiency (${E}_i^w$) in distinct frequency bands revealed the alterations of nodal efficiency in both patient groups (Fig. 4).

Compared to HC, the regions with the most significant group effects were mainly concentrated in IMF3 and predominantly observed in the visual cortex in the NDPD group (Fig. 4a). Decreased ${E}_i^w$ of IMF3 in the NDPD group was observed in the bilateral lingual gyri (LING), superior occipital gyrus (SOG), left postcentral gyri (PoCG), right calcarine fissure (CAL), cuneus (CUN), middle occipital gyrus (MOG), fusiform gyrus (FFG) and angular gyrus (ANG), while increased ${E}_i^w$ of component IMF4 was observed in the left supplementary motor area (SMA) (Fig. 4a and Table 4).

**Table 4:** Between-group differences of nodal efficiency among NDPD, DPD and HC

		Regions	Functional classification	Anatomical classification	Difference values (P values)
HC vs NDPD	IMF1	NS	NS	NS	NS
	IMF2	NS	NS	NS	NS
	IMF3	CAL.R	Primary	Occipital	3.09 (0.0027)
		CUN.R	Association	Occipital	2.92 (0.0044)
		LING.L	Association	Occipital	2.72 (0.0079)
		LING.R	Association	Occipital	3.14 (0.0023)
		SOG.L	Association	Occipital	3.16 (0.0022)
		SOG.R	Association	Occipital	3.25 (0.0016)
		MOG.R	Association	Occipital	4.34 (0.00004)
		FFG.R	Association	Temporal	3.00 (0.0035)
		PoCG.L	Primary	Parietal	2.71 (0.0081)
		ANG.R	Association	Parietal	2.94 (0.0041)
	IMF4	SMA.L	Association	Frontal	-3.42 (0.0009)
	IMF5	NS	NS	NS	NS
HC vs DPD	IMF1	ORBsup.L	Paralimbic	Prefontal	2.80 (0.0071)
	IMF2	PreCG.R	Primary	Frontal	3.18 (0.0023)
		ORBmid.L	Paralimbic	Prefontal	2.70 (0.0090)
		HIP.L	Limbic	Temporal	2.72 (0.0084)
		AMYG.R	Subcortical	Temporal	2.85 (0.0058)
		AMYG.L	Subcortical	Temporal	2.79 (0.0069)
	IMF3	ACG.L	Paralimbic	Prefontal	3.10 (0.0029)
		PoCG.L	Primary	Parietal	2.74 (0.0080)
		CAU.L	Subcortical	Subcortical	4.39 (0.00004)
		CAU.R	Subcortical	Subcortical	2.94 (0.0046)
	IMF4	SOG.R	Association	Occipital	2.94 (0.0045)
	IMF5	INS.L	Paralimbic	Subcortical	-2.77 (0.0073)
DPD vs NDPD	IMF1	NS	NS	NS	NS
	IMF2	NS	NS	NS	NS
	IMF3	CAU.R	Subcortical	Subcortical	-2.99 (0.0039)
		PUT.L	Subcortical	Subcortical	-2.68 (0.0093)
	IMF4	PUT.R	Subcortical	Subcortical	-3.15 (0.0025)
	IMF5	NS	NS	NS	NS

The functional connectivity networks for each participant in five specific brain oscillations were constructed using an AAL template. Pair wise comparisons were performed using a general linear model (adjusted for the effects of age, gender and years of education) to determine the inter-group differences for regional efficiency. A value of *P* < 0.01(uncorrected) was considered statistically significant. Only the maximum inter-group significant difference of regional efficiency in one IMF was reported specifically. NS: Not significant. For difference values, negative values in each pair-wise comparison represent HC < NDPD, HC < DPD, and DPD < NDPD respectively. *IMF* Intrinsic mode decomposition; *HC* healthy control; *NDPD* non-depressed Parkinson's disease; *DPD* depressed Parkinson's disease; the abbreviations of the regions, refer to Table 1

**Fig. 5:**
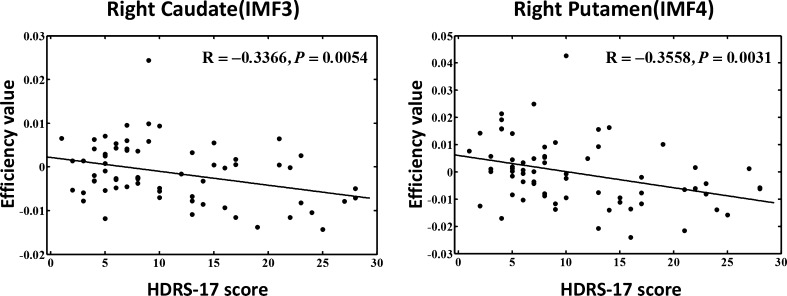
Relationship between frequency specific nodal efficiency and clinical variables. The results of the correlation analysis between the HDRS-17 score (x-axis) and frequency specific nodal efficiency in the left thalamus (R = -0.2721, *P* = 0.0259) and right caudate nucleus (R = -0.3366, *P* = 0.0054) in IMF3 component, and of the right putamen (R = -0.3558, *P* = 0.0031) and pallidum (R = -0.3184, *P* = 0.0086) in IMF4. *HDRS-17* 17-item Hamilton Depression Rating Scale; *IMF* Intrinsic mode decomposition

Relative to HC, the most significant observed group effects mainly focused on IMF2 and IMF3, and were predominately distributed in the paralimbic-limbic system in the DPD group (Fig. 4b). Decreased ${E}_i^w$ was observed in DPD patients in the left orbital part of superior frontal gyrus (ORBsup) from IMF1; bilateral amygdala (AMYG), left orbital part of middle frontal gyrus (ORBmid), hippocampus (HIP), and right precentral gyrus (PreCG) from IMF2; bilateral caudate nucleus (CAU), left anterior cingulate gyrus (ACG) and PoCG from IMF3; and right SOG from IMF4. Meanwhile, increased ${E}_i^w$ of component IMF5 was observed in the left insula (INS) (Fig. 4b and Table 4). Compared with NDPD, the most obvious observation for group effects was concentrated in IMF3 and IMF4 components and was distributed in the subcortical network in the DPD group (Fig. 4c). Decreased ${E}_i^w$ was observed in the right CAU and left putamen (PUT) from IMF3, and right PUT from IMF4 in the DPD group (Fig. 4c and Table 4). In addition, ${E}_i^w$ of the right CAU (R = -0.3366, *P* = 0.0054) in IMF3 and right PUT (R = -0.3558, *P* = 0.0031) in IMF4 were negatively correlated with the HDRS-17 score (Fig. 5).

## Discussion

In this current study with the utilization of resting state fMRI, the results suggested the dissociations of topological organizations of brain networks between NDPD and DPD patients in both spatial and spectral domains. Our data revealed three major findings: i) Compared to HC, the increased characteristic path length was exclusively observed in IMF2 in the DPD group and in IMF3 in the NDPD group; (ii) The nodes with significant topological alterations were mainly distributed in the visual cortex in NDPD patients, or in the paralimbic-limbic and basal ganglia networks in DPD patients at the nodal topological level; (iii) The significant disrupted nodal topological characteristic was dominated in the frequency band from 0.02 to 0.05 Hz in the visual cortex in the NDPD group , as well as from 0.01 to 0.05 Hz in the basal ganglia and from 0.02 to 0.22 Hz in the paralimbic-limbic network in the DPD group.

Global network measurements (i.e. γ and λ) indicated a small-world organization of all frequency specific brain networks in all groups. Compared to HC, the increased characteristic path length observed in IMF2 and IMF3 of the DPD and NDPD groups respectively, indicated an altered organization of the brain networks with specific brain oscillation leading to lower efficiency. Additionally, a previous electrophysiological study suggested that distinct motor and non-motor components of clinical impairment in Parkinson’s disease might be detected as oscillatory activities across multiple frequency bands and their cross-frequency interactions within spatially segregated loops of the basal ganglia-thalamo-cortical system (Oswal et al. 2013). Performing resting state fMRI, dissociation in global topological patterns of brain networks between NDPD and DPD patients in the frequency domain was observed. The results indicated the potential disassociation of global FC differences with their corresponding activities in distinct resting oscillation rhythms. In addition, the results of some topological profiles, including Cp in IMF2, and γ in IMF1 and IMF2, were difficult to interpret due to the fact that their inter-group differences were only significant at a narrow range of sparsity. Future studies may classify this topic in a large cohort.

Regarding regional properties, brain regions with reduced efficiency in NDPD were predominately located in the occipital cortex, which was consistent with previous studies (Gottlich et al. 2013; Luo et al. 2014; Skidmore et al. 2011). Previous studies documented that the Parkinson’s disease-related visual damages ranged from deficits in basic perceptual and semantic visual processing at an early stage of cognitive deterioration (Laatu et al. 2004; Cardoso et al. 2010), problems in motion and orientation discrimination (Bodis-Wollner and Paulus 1999; Trick et al. 1994) to even visual hallucinations (Barnes and David 2001; Mindham 2001). The observed modification of nodal efficiency in the right ANG might account for the non-motor symptoms of patients with Parkinson’s disease such as linguistic deficits and executive dysfunction (Altmann and Troche 2011; Kudlicka et al. 2011; Murray and Rutledge 2014; Shirer et al. 2012). Meanwhile, decreased efficiency in the left PoCG might be associated with the motor symptoms in Parkinson’s disease, which was consistent with a previous study (Sharman et al. 2013). Similarly, the disrupted BOLD signal activities in the PoCG and PreCG were also observed in the DPD group. On the other hand, a higher efficiency observed in the left supplementary motor area might reflect the compensatory mechanisms in Parkinson’s disease (Kwak et al. 2010; Yu et al. 2013; Sharman et al. 2013).

The occurrence of depression in Parkinson’s disease might arise from the disturbance in the paralimbic-limbic system as well as the basal ganglia network. Our results highlighted the critical role of nodes in the paralimbic-limbic system in DPD, which was consistent with previous studies (Luo et al. 2014; Skidmore et al. 2013). In addition, extensive evidence indicated that the abnormal function of the striatum and the associated limbic-basal ganglia circuitry played a role in the emotional processing system in patients with depression (Jiao et al. 2011; Lui et al. 2011; Marchand et al. 2012). Moreover, a recent study reported by Joutsa et al. (2013) suggested that impaired striatal dopaminergic function was related to depression symptoms in Parkinson’s disease. In the current study, decreased nodal efficiency observed in DPD patients in bilateral CAU (compared with HC) as well as bilateral PUT and right CAU (compared with NDPD) further supported the hypothesis. Additionally, the increased regional efficiencies in the left INS observed in DPD patients might have resulted from depression symptoms in Parkinson’s disease. For instance, recent studies (Connolly et al. 2013; Frodl et al. 2010) indicated an increased FC between the subgenual anterior cingulate and insula cortex in patients with depression, which goes along with our findings that the subgenual anterior cingulate was likely to be the vital hubs in the neural circuits associated with depression in Parkinson’s disease. It was disputable whether regional efficiency differences demonstrated by fMRI in the high frequency band (IMF1, 0.12~0.22 Hz) were attributable to a susceptibility artifact as the frequency band such as the IMF1 was covered by a respiratory frequency interval from 0.1 to 0.5 Hz (Cordes et al. 2001). However, several other studies detected that the spectral range of BOLD signals was greater than 0.1 Hz, which also demonstrated consistent patterns with low-frequency fluctuations (< 0.1 Hz) (Boubela et al. 2013; Van Someren 2011). Furthermore, the orbitofrontal cortex detected in IMF1 might be the key region in DPD (Choe et al. 2013; Gottlich et al. 2013). Hence, we speculated that the high frequency band IMF1 might be associated with some physiological significance, rather than susceptibility artifact.

Meanwhile, these nodes with significant group effects were dominated in distinct frequency bands. From one perspective, it has been suggested that the brain oscillations within distinct frequency bands were generated by diverse mechanisms and possessed different physiological functions (Buzsaki and Draguhn 2004; Engel et al. 2001; Penttonen and Buzsáki 2003). In addition, the previous study (Oswal et al. 2013) suggested the associations between the distinct functional circuits oscillatory activities across multiple frequency bands and distinct motor, non-motor components of clinical impairment in Parkinson’s disease. From another perspective, the distinct frequency characteristics of spatially distributed nodes were complicated, concerning unclarified origins, relations, and specific physiological functions of different oscillatory bands. Future work combining EEG, resting state fMRI as well as various task-based fMRI may contribute to a more precise understanding of the neurophysiological basis of the signals located in different frequency bands.

### Limitations

In the current study, the comparison of frequency-specific brain networks was performed. Several limitations were noteworthy. The first limitation was due to the little-known influence of head motion on the frequency specificities in a small world network, since several recent studies (Power et al. 2012; Van Dijk et al. 2012) reported decreased long range connectivity and increased local connectivity due to head motion. Further investigation will be conducted in our next study. Moreover, other methods were not considered to define the value of FC, like partial correlation, which could estimate the direct interdependence after ruling out third-party effects (Pereda et al. 2005). Another limitation of our study was that the analysis of node definition was limited to AAL template-based brain networks. A previous study suggested that the topological organization of brain networks was affected by various parcellation strategies (Wang et al. 2009). Future studies need to clarify the impact of various node definitions on this topic. Additionally, in the FC analysis in BOLD fMRI, an ongoing debate on the necessity to correct global signals in fMRI time courses did not reach a consensus (Hayasaka 2013). One previous study (Hayasaka 2013) suggested that without global signal correction, nodes along the inter-hemispheric fissure were highly connected while some nodes and subgraphs around white-matter tracts became disconnected from the rest of the network. In the current study, regression of global signals was performed. The discussion on how this preprocessing step influenced the results was beyond our focus. Lastly, the conclusion of the current study was demonstrated using limited sample data; further studies need to be done in a large cohort.

## Conclusion

In the present study, we have introduced a novel method CEEMD to divide the resting state fMRI signals into five specific brain oscillations within distinct frequency bands. Our results demonstrated that brain networks for all groups showed small world architecture, and the optimal topological organization of brain networks decreased in both NDPD and DPD patients. Moreover, disrupted global topological organization, Lp, was distinct from frequency between NDPD and DPD groups. Additionally, altered brain regions within three key neural networks in NDPD and DPD patients and distinct frequency characteristics of these spatially distributed nodes were demonstrated. Overall, the present study demonstrated that there was dissociation in topological organizations of brain networks between NDPD and DPD patients in spatial and frequency domains. More importantly, the initial method described in the current study provided a novel insight to investigate the neuroimaging biomarkers of various brain disorders in both spatial and spectral domains.

## Electronic supplementary material

Supplementary Table 1 (DOCX 15 kb)

Supplementary Table 2 (DOCX 15 kb)

Supplementary Table 3 (DOCX 15 kb)

Supplementary Table 4 (DOCX 15 kb)

Supplementary Table 5 (DOCX 15 kb)

Supplementary Figure 1 **The distribution of hub regions in the HC, NDPD and DPD groups in each frequency band.** Hub regions were visualized using BrainNet view (NKLCNL, Beijing Normal University). Three dimensional rendering maps showed the hub regions defined by normalized nodal efficiency in each frequency band as described by Zhang et al. (2011) (Supplementary Table 1 to 5). The hub nodes are shown in red, yellow, cyan, and blue colors represent the Associations, Primary, Paralimbic and Limbic regions. The abbreviations of the regions are shown in Table 1. HC = Healthy control; NDPD = Non-depressed Parkinson's disease; DPD = Depressed Parkinson's disease. (GIF 161 kb)

High resolution image (TIFF 6485 kb)
