# Supplementary material for: Frequency specific brain networks in Parkinson’s disease and comorbid depression
Source: Brain Imaging Behav. 2016 Feb 5;11(1):224–39. doi: 10.1007/s11682-016-9514-9 (PMC5415593; doi:10.1007/s11682-016-9514-9)
Supplement: Supplementary file 1 — (DOCX 15 kb) [file 11682_2016_9514_MOESM1_ESM.docx]

**Supplementary Table 1: Hub Regions of FC networks among HC, NDPD and DPD groups in IMF1.**

| IMF1 | Hub regions | class | E_nodal_/mean |
| --- | --- | --- | --- |
| HC | ORBsupmed.R | Paralimbic | 1.1448 |
|  | ORBsupmed.L | Paralimbic | 1.1412 |
|  | SOG.L | Association | 1.0878 |
|  | MOG.L | Association | 1.0837 |
|  | ACG.L | Paralimbic | 1.0828 |
|  | REC.L | Paralimbic | 1.0818 |
|  | SOG.R | Association | 1.0815 |
|  | ACG.R | Paralimbic | 1.0797 |
|  | ORBinf.L | Paralimbic | 1.0761 |
|  | SFGmed.R | Association | 1.0718 |
|  | SFGmed.L | Association | 1.0698 |
|  | CUN.R | Association | 1.0688 |
|  | STG.R | Association | 1.0673 |
|  | ORBinf.R | Paralimbic | 1.0645 |
| NDPD | ORBsupmed.R | Paralimbic | 1.1080 |
|  | SFGmed.L | Association | 1.1006 |
|  | IPL.L | Association | 1.0921 |
|  | SFGdor.L | Association | 1.0878 |
|  | ORBsupmed.L | Paralimbic | 1.0851 |
|  | ACG.L | Paralimbic | 1.0819 |
|  | ACG.R | Paralimbic | 1.0746 |
|  | SFGdor.R | Association | 1.0735 |
|  | SFGmed.R | Association | 1.0717 |
|  | STG.L | Association | 1.0673 |
|  | TPOsup.L | Paralimbic | 1.0658 |
|  | MFG.L | Association | 1.0649 |
|  | MFG.R | Association | 1.0624 |
|  | MOG.L | Association | 1.0616 |
| DPD | ORBsupmed.R | Paralimbic | 1.1239 |
|  | IPL.L | Association | 1.1186 |
|  | CAL.L | Primary | 1.1054 |
|  | SFGmed.L | Association | 1.1051 |
|  | STG.L | Association | 1.1004 |
|  | MOG.L | Association | 1.0911 |
|  | ORBsupmed.L | Paralimbic | 1.0899 |
|  | SOG.L | Association | 1.0877 |
|  | SFGdor.L | Association | 1.0853 |
|  | CUN.R | Association | 1.0835 |
|  | MTG.L | Association | 1.0788 |
|  | REC.L | Paralimbic | 1.0711 |
|  | ANG.L | Association | 1.0698 |
