# Supplementary material for: Frequency specific brain networks in Parkinson’s disease and comorbid depression
Source: Brain Imaging Behav. 2016 Feb 5;11(1):224–39. doi: 10.1007/s11682-016-9514-9 (PMC5415593; doi:10.1007/s11682-016-9514-9)
Supplement: Supplementary file 2 — (DOCX 15 kb) [file 11682_2016_9514_MOESM2_ESM.docx]

**Supplementary Table 2: Hub regions of FC networks among HC, NDPD and DPD groups in IMF2.**

| IMF2 | Hub regions | class | E_nodal_/mean |
| --- | --- | --- | --- |
| HC | ORBsupmed.L | Paralimbic | 1.1196 |
|  | STG.L | Association | 1.1113 |
|  | ORBsupmed.R | Paralimbic | 1.1110 |
|  | TPOsup.R | Paralimbic | 1.0970 |
|  | ACG.L | Paralimbic | 1.0956 |
|  | REC.L | Paralimbic | 1.0911 |
|  | SFGmed.L | Association | 1.0895 |
|  | STG.R | Association | 1.0827 |
|  | TPOsup.L | Paralimbic | 1.0748 |
|  | ACG.R | Paralimbic | 1.0702 |
|  | ORBinf.L | Paralimbic | 1.0697 |
|  | REC.R | Paralimbic | 1.0650 |
| NDPD | ORBsupmed.L | Paralimbic | 1.1235 |
|  | SFGmed.L | Association | 1.1120 |
|  | ORBsupmed.R | Paralimbic | 1.1095 |
|  | ACG.L | Paralimbic | 1.1079 |
|  | ACG.R | Paralimbic | 1.0939 |
|  | SFGmed.R | Association | 1.0896 |
|  | SFGdor.L | Association | 1.0875 |
|  | IFGtriang.L | Association | 1.0854 |
|  | STG.L | Association | 1.0717 |
|  | IFGoperc.L | Association | 1.0678 |
|  | IPL.L | Association | 1.0646 |
|  | PreCG.L | Primary | 1.0632 |
|  | TPOsup.L | Paralimbic | 1.0619 |
|  | REC.L | Paralimbic | 1.0617 |
| DPD | ORBsupmed.L | Paralimbic | 1.1444 |
|  | ORBsupmed.R | Paralimbic | 1.1243 |
|  | SFGmed.L | Association | 1.1224 |
|  | REC.L | Paralimbic | 1.1214 |
|  | CAL.L | Primary | 1.1073 |
|  | SFGdor.L | Association | 1.0930 |
|  | CAL.R | Primary | 1.0905 |
|  | STG.L | Association | 1.0882 |
|  | REC.R | Paralimbic | 1.0804 |
|  | ANG.L | Association | 1.0798 |
|  | PCG.L | Paralimbic | 1.0747 |
|  | CUN.L | Association | 1.0695 |
|  | ACG.L | Paralimbic | 1.0671 |
