# Supplementary material for: Frequency specific brain networks in Parkinson’s disease and comorbid depression
Source: Brain Imaging Behav. 2016 Feb 5;11(1):224–39. doi: 10.1007/s11682-016-9514-9 (PMC5415593; doi:10.1007/s11682-016-9514-9)
Supplement: Supplementary file 3 — (DOCX 15 kb) [file 11682_2016_9514_MOESM3_ESM.docx]

**Supplementary Table 3: Hub regions of FC networks among HC, NDPD and DPD groups in IMF3.**

| IMF3 | Hub regions | class | E_nodal_/mean |
| --- | --- | --- | --- |
| HC | ORBsupmed.R | Paralimbic | 1.0948 |
|  | ORBsupmed.L | Paralimbic | 1.0902 |
|  | REC.R | Paralimbic | 1.0810 |
|  | REC.L | Paralimbic | 1.0735 |
|  | SOG.R | Association | 1.0731 |
|  | STG.L | Association | 1.0706 |
|  | LING.R | Association | 1.0701 |
|  | ACG.L | Paralimbic | 1.0620 |
|  | ORBinf.R | Paralimbic | 1.0596 |
|  | SFGmed.L | Association | 1.0591 |
|  | LING.L | Association | 1.0588 |
|  | SOG.L | Association | 1.0580 |
|  | CAL.R | Primary | 1.0577 |
|  | SFGmed.R | Association | 1.0522 |
|  | INS.R | Paralimbic | 1.0516 |
|  | IFGtriang.R | Association | 1.0503 |
|  | ACG.R | Paralimbic | 1.0497 |
|  | CUN.R | Association | 1.0496 |
| NDPD | SFGmed.L | Association | 1.1038 |
|  | ORBsupmed.L | Paralimbic | 1.0918 |
|  | ORBsupmed.R | Paralimbic | 1.0878 |
|  | REC.L | Paralimbic | 1.0833 |
|  | INS.R | Paralimbic | 1.0812 |
|  | TPOsup.R | Paralimbic | 1.0731 |
|  | REC.R | Paralimbic | 1.0724 |
|  | STG.L | Association | 1.0693 |
|  | PCG.L | Paralimbic | 1.0635 |
|  | SFGmed.R | Association | 1.0571 |
|  | ACG.L | Paralimbic | 1.0566 |
|  | TPOsup.L | Paralimbic | 1.0560 |
|  | ACG.R | Paralimbic | 1.0548 |
|  | SFGdor.L | Association | 1.0545 |
|  | STG.R | Association | 1.0541 |
| DPD | REC.L | Paralimbic | 1.1348 |
|  | ORBsupmed.L | Paralimbic | 1.1210 |
|  | REC.R | Paralimbic | 1.1170 |
|  | ORBsupmed.R | Paralimbic | 1.1049 |
|  | SFGmed.L | Association | 1.0931 |
|  | TPOsup.R | Paralimbic | 1.0760 |
|  | STG.L | Association | 1.0717 |
|  | LING.R | Association | 1.0601 |
|  | CAL.L | Primary | 1.0593 |
