# Supplementary material for: Frequency specific brain networks in Parkinson’s disease and comorbid depression
Source: Brain Imaging Behav. 2016 Feb 5;11(1):224–39. doi: 10.1007/s11682-016-9514-9 (PMC5415593; doi:10.1007/s11682-016-9514-9)
Supplement: Supplementary file 4 — (DOCX 15 kb) [file 11682_2016_9514_MOESM4_ESM.docx]

**Supplementary Table 4: Hub regions of FC networks among HC, NDPD and DPD groups in IMF4.**

| IMF4 | Hub regions | class | E_nodal_/mean |
| --- | --- | --- | --- |
| HC | STG.L | Association | 1.0922 |
|  | SOG.R | Association | 1.0834 |
|  | REC.R | Paralimbic | 1.0743 |
|  | STG.R | Association | 1.0702 |
|  | ORBsupmed.R | Paralimbic | 1.0700 |
|  | SOG.L | Association | 1.0646 |
|  | REC.L | Paralimbic | 1.0559 |
|  | ORBsupmed.L | Paralimbic | 1.0529 |
|  | PoCG.L | Primary | 1.0516 |
|  | MOG.R | Association | 1.0485 |
|  | TPOsup.R | Paralimbic | 1.0453 |
|  | CUN.R | Association | 1.0396 |
|  | OLF.R | Limbic | 1.0379 |
|  | PoCG.R | Primary | 1.0371 |
| NDPD | REC.L | Paralimbic | 1.0963 |
|  | REC.R | Paralimbic | 1.0817 |
|  | SFGmed.L | Association | 1.0737 |
|  | STG.R | Association | 1.0712 |
|  | SFGmed.R | Association | 1.0702 |
|  | ORBsupmed.R | Paralimbic | 1.0681 |
|  | STG.L | Association | 1.0656 |
|  | ORBsupmed.L | Paralimbic | 1.0628 |
|  | ACG.R | Paralimbic | 1.0550 |
|  | ACG.L | Paralimbic | 1.0483 |
|  | TPOsup.R | Paralimbic | 1.0471 |
|  | PCG.L | Paralimbic | 1.0399 |
|  | INS.R | Paralimbic | 1.0387 |
| DPD | STG.L | Association | 1.1166 |
|  | REC.L | Paralimbic | 1.1064 |
|  | ORBsupmed.L | Paralimbic | 1.1022 |
|  | ORBsupmed.R | Paralimbic | 1.0891 |
|  | TPOsup.L | Paralimbic | 1.0778 |
|  | PCG.L | Paralimbic | 1.0778 |
|  | INS.L | Paralimbic | 1.0750 |
|  | ORBinf.L | Paralimbic | 1.0636 |
|  | REC.R | Paralimbic | 1.0630 |
|  | STG.R | Association | 1.0580 |
|  | INS.R | Paralimbic | 1.0568 |
|  | SFGmed.L | Association | 1.0540 |
|  | IFGtriang.L | Association | 1.0479 |
|  | TPOsup.R | Paralimbic | 1.0464 |
