# Supplementary material for: Frequency specific brain networks in Parkinson’s disease and comorbid depression
Source: Brain Imaging Behav. 2016 Feb 5;11(1):224–39. doi: 10.1007/s11682-016-9514-9 (PMC5415593; doi:10.1007/s11682-016-9514-9)
Supplement: Supplementary file 5 — (DOCX 15 kb) [file 11682_2016_9514_MOESM5_ESM.docx]

**Supplementary Table 5: Hub regions of FC networks among HC, NDPD and DPD groups in IMF5.**

| IMF5 | Hub regions | class | E_nodal_/mean |
| --- | --- | --- | --- |
| HC | MOG.R | Association | 1.0839 |
|  | SOG.R | Association | 1.0758 |
|  | SOG.L | Association | 1.0753 |
|  | PoCG.L | Primary | 1.0747 |
|  | STG.L | Association | 1.0741 |
|  | PHG.R | Paralimbic | 1.0697 |
|  | CUN.R | Association | 1.0633 |
|  | PHG.L | Paralimbic | 1.0596 |
|  | TPOmid.L | Paralimbic | 1.0533 |
|  | STG.R | Association | 1.0532 |
|  | OLF.L | Limbic | 1.0503 |
|  | REC.R | Paralimbic | 1.0500 |
|  | AMYG.R | Subcortical | 1.0458 |
|  | SMG.R | Association | 1.0445 |
|  | CUN.L | Association | 1.0422 |
| NDPD | REC.R | Paralimbic | 1.0743 |
|  | TPOmid.R | Paralimbic | 1.0631 |
|  | STG.L | Association | 1.0568 |
|  | REC.L | Paralimbic | 1.0531 |
|  | STG.R | Association | 1.0480 |
|  | ACG.L | Paralimbic | 1.0479 |
|  | PreCG.R | Primary | 1.0433 |
|  | PHG.L | Paralimbic | 1.0426 |
|  | SFGmed.L | Association | 1.0426 |
|  | OLF.L | Limbic | 1.0421 |
|  | ORBsupmed.R | Paralimbic | 1.0421 |
|  | TPOmid.L | Paralimbic | 1.0387 |
|  | PHG.R | Paralimbic | 1.0328 |
|  | SOG.R | Association | 1.0318 |
|  | SFGmed.R | Association | 1.0311 |
|  | CAL.L | Primary | 1.0303 |
| DPD | TPOmid.L | Paralimbic | 1.1053 |
|  | INS.L | Paralimbic | 1.1040 |
|  | OLF.L | Limbic | 1.0877 |
|  | STG.L | Association | 1.0840 |
|  | TPOsup.R | Paralimbic | 1.0816 |
|  | PHG.L | Paralimbic | 1.0732 |
|  | INS.R | Paralimbic | 1.0689 |
|  | OLF.R | Limbic | 1.0679 |
|  | REC.R | Paralimbic | 1.0650 |
|  | TPOmid.R | Paralimbic | 1.0621 |
|  | ORBsup.R | Paralimbic | 1.0617 |
|  | HIP.R | Limbic | 1.0617 |
|  | ROL.L | Association | 1.0612 |
