# Supplementary figures and images for: Frequency specific brain networks in Parkinson’s disease and comorbid depression
Source: Brain Imaging Behav. 2016 Feb 5;11(1):224–39. doi: 10.1007/s11682-016-9514-9 (PMC5415593; doi:10.1007/s11682-016-9514-9)

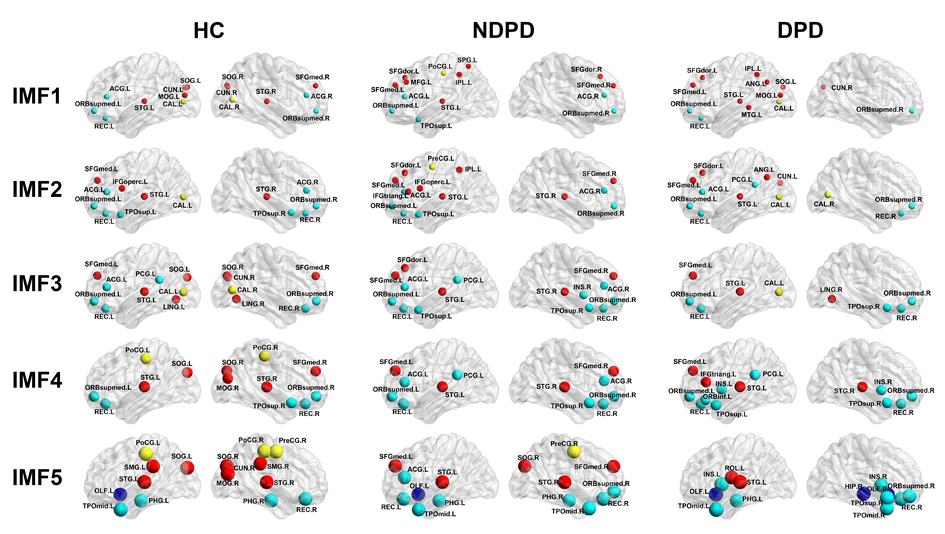

Supplement: Supplementary file 6 — The distribution of hub regions in the HC, NDPD and DPD groups in each frequency band. Hub regions were visualized using BrainNet view (NKLCNL, Beijing Normal University). Three dimensional rendering maps showed the hub regions defined by normalized nodal efficiency in each frequency band as described by Zhang et al. (2011) (Supplementary Table 1 to 5). The hub nodes are shown in red, yellow, cyan, and blue colors represent the Associations, Primary, Paralimbic and Limbic regions. The abbreviations of the regions are shown in Table 1. HC = Healthy control; NDPD = Non-depressed Parkinson's disease; DPD = Depressed Parkinson's disease. (GIF 161 kb) [file 11682_2016_9514_Fig7_ESM.gif]

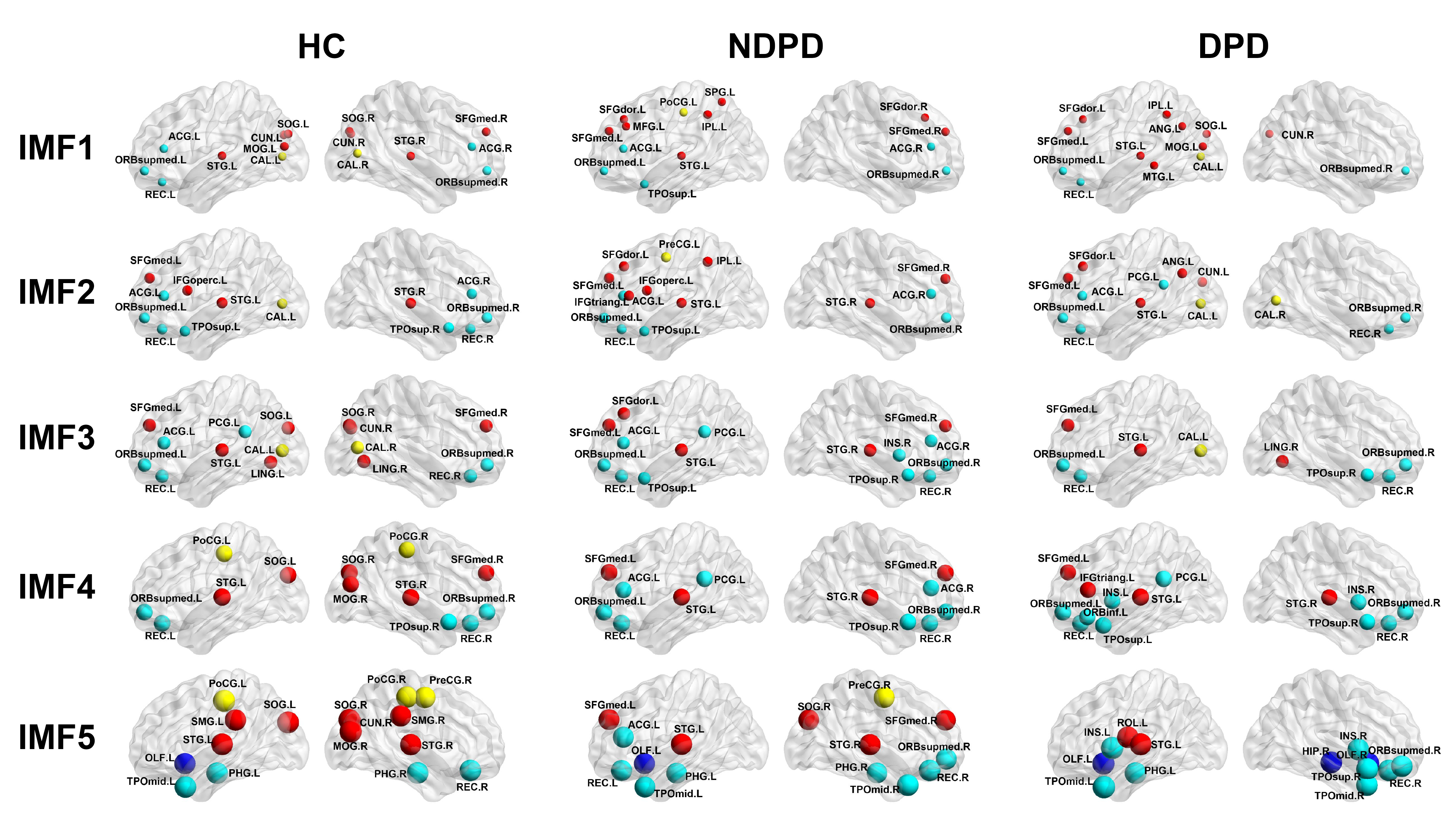

Supplement: Supplementary file 7 — High resolution image (TIFF 6485 kb) [file 11682_2016_9514_MOESM6_ESM.tif]
